# Diagnostic value of superb microvascular imaging in cardiac metastasis

**DOI:** 10.1093/ehjimp/qyaf114

**Published:** 2025-09-06

**Authors:** Yuta Torii, Madoka Sano, Hideyuki Hayashi, Aoi Hamano, Yutaka Furukawa

**Affiliations:** Department of Clinical Laboratory, Kobe City Medical Center General Hospital, 2-1-1 Minatojima Minamimachi, Chuo-ku, Kobe, Hyogo 650-0047, Japan; Department of Cardiovascular Medicine, Kobe City Medical Center General Hospital, Kobe, Hyogo, Japan; Department of Cardiovascular Medicine, Kobe City Medical Center General Hospital, Kobe, Hyogo, Japan; Department of Clinical Laboratory, Kobe City Medical Center General Hospital, 2-1-1 Minatojima Minamimachi, Chuo-ku, Kobe, Hyogo 650-0047, Japan; Department of Cardiovascular Medicine, Kobe City Medical Center General Hospital, Kobe, Hyogo, Japan

**Keywords:** neoplasm metastasis, superb microvascular imaging, coronary flow

A 90-year-old woman with advanced right upper lobe lung carcinoma managed with best supportive care presented with acute dyspnoea and chest pain. Electrocardiography showed ST-segment elevation in leads II, III, aVF, V5, and V6, with reciprocal depression in V1–V3 (*Figure 1A*), and cardiac troponin I was elevated (0.397 ng/mL), findings suggestive of ST-segment elevation myocardial infarction (STEMI). Transthoracic echocardiography demonstrated a heterogeneous mass infiltrating the lateral left ventricular wall, contiguous with the pericardium (*Figure 1B*). Conventional colour Doppler imaging was inadequate, particularly for assessing the left circumflex territory (see Supplementary data online, *Video S1*; frame rates in the caption). Superb microvascular imaging (SMI; Aplio i700, Canon) delineated preserved intramyocardial microvascular flow in the left circumflex (LCx) territory traversing the mass towards the apex (*Figure 1C*), while Doppler sampling showed systolic flow reversal with steep early-diastolic forward flow—features compatible with extrinsic compression rather than flow-limiting occlusion (*Figure 1D*). Given her frailty and goals of care, angiography was not pursued. The working diagnosis was ST-segment elevation secondary to myocardial infiltration with LCx compression by metastatic tumour, rather than plaque rupture STEMI. Serial electrocardiograms (ECGs) over 72 h showed no evolutionary change (*Figure 1E*), and biomarker trajectory—with troponin I reaching 0.627 ng/mL—together supported a non-ischaemic mechanism. The patient was managed conservatively with goal-concordant care. SMI changed management by non-invasively demonstrating preserved LCx territory flow and a compression haemodynamic pattern when colour Doppler was non-diagnostic, thereby avoiding urgent angiography. This case underscores the incremental value of SMI for LCx territory assessment in cancer-related cardiac masses presenting with apparent STEMI.

**Figure 1:**
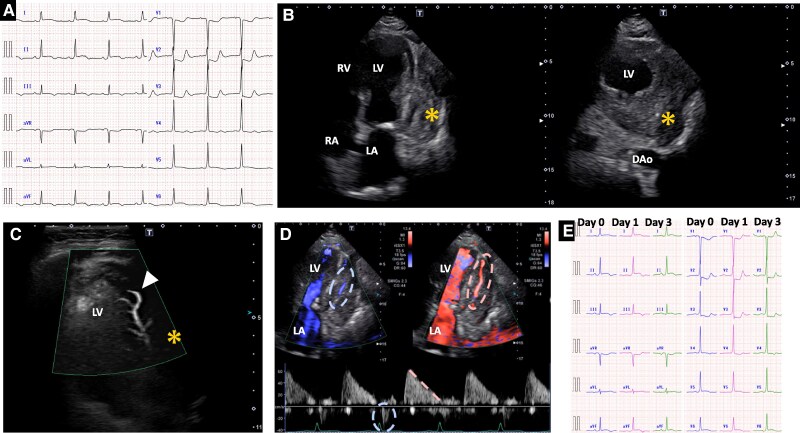
(*A*) Twelve-lead ECG on admission showing ST-segment elevation in leads II, III, aVF, and V5–V6 with reciprocal depression in V1–V3. (*B*) Transthoracic echocardiographic (TTE) images (apical four-chamber and parasternal short-axis views) show a heterogeneous mass infiltrating the LV lateral/inferolateral wall, contiguous with the pericardium. Asterisk: cardiac tumour. Orientation labels: LV, LA, RV, and DAo. (*C*) SMI (apical four-chamber) depicts preserved LCx territory flow (white arrowheads) traversing the mass towards the apex, indicating no haemodynamically significant coronary occlusion. Asterisk: cardiac tumour. (*D*) Colour-coded SMI Doppler in the apical four-chamber view by transthoracic echocardiography at the inferolateral wall shows systolic reversed flow (blue circle) and steep diastolic forward flow (red circle), consistent with extrinsic compression. (*E*) Serial ECGs on Day 0, Day 1, and Day 3 demonstrating no evolutionary changes.

## Supplementary data

Supplementary data are available at *European Heart Journal - Imaging Methods and Practice* online.

**Funding:** None declared.

## Ethical statements

All procedures followed were in accordance with the ethical standards of the responsible committee on human experimentation (institutional and national) and with the Helsinki Declaration of 1964 and later versions. Informed consent for the submission and publication of this case report was obtained from the patient.

## Supplementary Material

qyaf114_Supplementary_Data

## Data Availability

No new data were generated or analysed in support of this research.

## Lead author biography

Yuta Torii, PhD, RDMS, received his doctoral degree in medicine from Tokushima University. He currently serves as a cardiac sonographer at Kobe City Medical Center General Hospital. His clinical and research interests focus on echocardiography and heart failure.

