# Supplementary figures and images for: Diagnostic value of superb microvascular imaging in cardiac metastasis
Source: Eur Heart J Imaging Methods Pract. 2025 Sep 6;3(2):qyaf114. doi: 10.1093/ehjimp/qyaf114 (PMC12451434; doi:10.1093/ehjimp/qyaf114)

## Slide 1
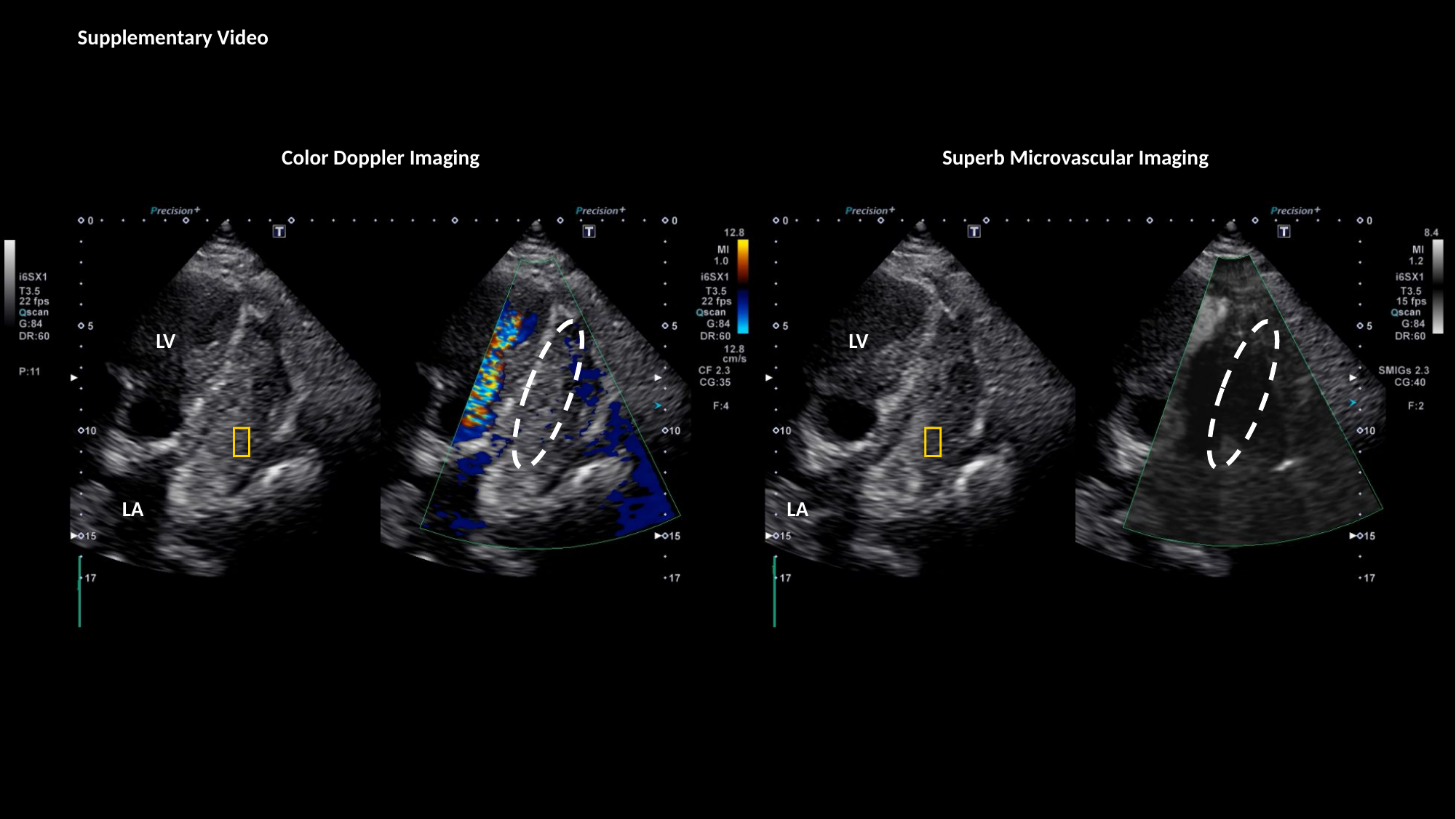

Supplementary Video
Color Doppler Imaging
Superb Microvascular Imaging
LV
LV
＊
＊
LA
LA

Supplement: qyaf114_Supplementary_Data [file qyaf114_supplementary_data.pptx]
